# The Longitudinal Impact of Hearing Impairment on Cognition Differs According to Cognitive Domain

**DOI:** 10.3389/fnagi.2016.00201

**Published:** 2016-08-22

**Authors:** Yasue Uchida, Yukiko Nishita, Chikako Tange, Saiko Sugiura, Rei Otsuka, Hiromi Ueda, Tsutomu Nakashima, Fujiko Ando, Hiroshi Shimokata

**Affiliations:** ^1^Department of Otorhinolaryngology, Aichi Medical UniversityNagakute, Aichi Prefecture, Japan; ^2^Department of Otorhinolaryngology, National Center for Geriatrics and GerontologyObu, Aichi Prefecture, Japan; ^3^Section of Longitudinal Study of Aging, National Institute for Longevity Sciences, National Center for Geriatrics and GerontologyObu, Aichi Prefecture, Japan; ^4^Ichinomiya Medical Treatment and Habilitation CenterIchinomiya, Aichi Prefecture, Japan; ^5^Faculty of Health and Medical Sciences, Aichi Shukutoku UniversityNagakute, Aichi Prefecture, Japan; ^6^Graduate School of Nutritional Sciences, Nagoya University of Art and ScienceNisshin, Aichi Prefecture, Japan

**Keywords:** hearing impairment, cognitive decline, longitudinal study, aging, Wechsler Adult Intelligence Scale, cognitive domain

## Abstract

Identification and modification of the risk factors for cognitive decline throughout the adult life span are priority subjects in a progressively aging society; however, much remains to be learned. The aim of this study was to understand whether changes in cognitive function can be affected by hearing impairment (HI) and whether the impact of HI differs depending on the cognitive domain. A total of 1109 individuals aged 60–79 years at baseline who participated in the Longitudinal Study of Aging at the National Institute for Longevity Sciences (NILS-LSA) was followed up for a maximum of 13.3 years. Cognitive function was evaluated using four subtests of the Japanese Wechsler Adult Intelligence Scale-Revised Short Forms (JWAIS-R-SF): namely, Information, Similarities, Picture Completion, and the Digit Symbol Substitution subtests. The HI was defined as a pure-tone average of the better ear >25 dB. A longitudinal analysis of 4437 observations obtained during a follow-up period of approximately 12 years was performed. We estimated linear changes in subtest scores by HI status, using the time-varying mixed-effects regression model, which included fixed terms for the intercept, HI status at baseline, time (years elapsed since baseline) and an HI × time interaction term adjusted for age at baseline, sex, education, and other possible confounders. There were significant main effects of HI on the scores of the four subtests after adjustment. The HI × time interaction was significant for the scores of the Information (*p* = 0.001) and Digit Symbol Substitution subtests (*p* = 0.001). The scores of the HI group declined faster in the Information and Digit Symbol Substitution subtests compared to those in the no-HI group. The model-predicted 12-year slope using a mean baseline age (68.7 years) indicated no significant decline in the individuals without HI at baseline for the Information and Similarities subtests, however, this tolerance was lost in the individuals with HI. In conclusion, the present observation showed that the impact of HI on cognition was longitudinally significant and implied that the effect differs according to cognitive domain.

## Introduction

As human life expectancy increases, the elderly population grows, and the number of people with cognitive impairment is expected to rise. Preventing individuals’ cognitive impairment and promoting their cognitive health are priority subjects in a progressively aging society. An advanced understanding of various exogenous factors contributes substantially to reducing risks and developing interventions; however, much remains to be learned about the modifiable risk factors for cognitive decline throughout the adult life span.

Presbycusis, also known as age-related hearing impairment (HI), is one of the most common chronic conditions in older adults. It is prevalence in one among three adults older than 65 years according to global estimates by the World Health Organization (WHO; WHO, 2012). Recently, a growing body of research has focused on the relationship between HI and cognitive decline. Multiple epidemiologic studies have shown the link between hearing and cognitive problems or incident dementia (Lin, 2011; Lin et al., 2011, 2014; Sugawara et al., 2011; Kiely et al., 2012; Gurgel et al., 2014; Moore et al., 2014; Quaranta et al., 2014; Dawes et al., 2015; Harrison Bush et al., 2015). Across studies using a wide variety of approaches and designs, it is likely that the differences in the methods used for hearing and cognitive assessments, the study population, age range, sample size, and cross-sectional (Lin, 2011; Sugawara et al., 2011; Moore et al., 2014; Quaranta et al., 2014; Dawes et al., 2015; Harrison Bush et al., 2015) or longitudinal (Lin et al., 2011, 2014; Kiely et al., 2012; Gurgel et al., 2014) analyses have an impact on the difference in outcomes.

Although all aging humans will develop some degree of decline in cognitive capacity, age-related changes in cognitive and intellectual abilities are not homogeneous (Ardila, 2007; Hartshorne and Germine, 2015). There are wide variations in the magnitude and rate depending on the specific cognitive function; in other words, some cognitive abilities stabilize and others may even improve over lifetime. A number of theories exist that elucidate the possible mechanisms that drive age-related changes in cognitive abilities (Horn and Cattell, 1967; McGillivray et al., 2012).

There are many factor models of cognition that describe the structure of intellectual functioning. The Cattell-Horn-Carroll (CHC) theory of cognitive abilities, which combines the Cattell-Horn Fluid-Crystallized (Gf-Gc) and Carroll three-stratum models, is one of the most widely accepted theories (Horn and Cattell, 1966; Carroll, 1993; Willis et al., 2011; Flanagan and Harrison, 2012; Schneider and Mcgrew, 2012). Significant age-related changes in cognition can occur in multiple domains. The fluid abilities are the most affected; in particular, episodic memory, processing speed, executive functions and working memory capacity suffer a substantial decline as people get older. In contrast, some aspects of cognition, such as crystallized intelligence and emotion regulation, tend to be maintained. Others propose that cognitive aging is more complex than the fluid-/crystalized-intelligence distinction suggests (Tucker-Drob, 2009; Hartshorne and Germine, 2015).

Previously, we investigated whether education level was associated with the degree of intellectual change among community-dwelling elder Japanese people during a follow-up of approximately 10 years, which was the same cohort as in the present analysis. The effect of educational level on 10-year changes in cognitive abilities differed according to specific functions (Nishita et al., 2013). As expected, individuals with higher levels of education had higher cognitive ability at every point in time; however, surprisingly, individuals with higher levels of education experienced a greater decline in the Digit Symbol Substitution subtest scores than those with less education over time. Education did not affect 10-year changes of the Information, Similarities and Picture Completion subtest scores. Although education is one of the most investigated modifiers of adult cognitive abilities, higher education did not show the protective effect against cognitive decline in our previous analysis. In the present study, we intended to clarify whether changes in cognitive abilities can be affected by HI and whether the impact of HI differs depending on the cognitive domain in a community-living elderly Japanese population.

## Materials and Methods

### Study Cohort at Baseline and Protocol

The participants in the present analyses were enrolled from the National Institute for Longevity Sciences—Longitudinal Study of Aging (NILS-LSA), a population-based biennial survey of a cohort of approximately 2200 adults. The NILS-LSA is a comprehensive and interdisciplinary study that observes age-related changes using detailed questionnaires and medical checkups, blood chemical analysis, body composition, anthropometry, physical function, nutritional analysis, psychological tests and tests of visual and auditory functions. The NILS-LSA participants were community-dwellers in Aichi Prefecture in central Japan who were randomly selected from resident registrations and stratified by both age and sex in cooperation with the local government. The number of men and women recruited were similar, and age at first-wave examination was 40 years to 79 years, with similar number of participants in each decade of age (i.e., 40, 50, 60, and 70 s). Participants have been followed up every 2 years. The examination intervals were as follows: first (November 1997–April 2000), second (April 2000–May 2002), third (May 2002–May 2004), fourth (June 2004–July 2006), fifth (July 2006–July 2008), sixth (July 2008–July 2010), and seventh (July 2010–July 2012). The first-wave examination was treated as baseline for the present analyses, and the sample consisted of 2267 participants aged between 40 and 79 years at baseline. The study protocol was approved by the Committee of Ethics of Human Research of the National Center for Geriatrics and Gerontology (approval number #14, #52, #74). Written informed consent was obtained from all participants. The details of the NILS-LSA have been described elsewhere (Shimokata et al., 2000).

For the present analyses, an initial sample of individuals who were aged 60 years or older at baseline completed an audiometric test and cognitive function measures were assessed, and they participated more than once after the first wave was selected. Individuals were excluded who had a history of dementia at baseline or had a deficiency of any required information, such as confounders at baseline. Those unable to fulfill only a small part of the subtests of the cognitive function measures due to a vision problem, finger trembling, or other reasons were included. The final sample that consisted of 1109 participants aged 60–79 years at baseline was followed up for a maximum of 13.3 years. A total of 4437 observations from 1109 participants, which were compiled from the first-through the seventh-wave examination surveyed between 1997 and 2012, were used for the analyses. The mean number of repeated visits was 4.6.

### Neuropsychological Assessments

The most common test of intelligence in adults is the Wechsler Adult Intelligence Scale (WAIS). Cognitive ability was evaluated with a battery of four subtests from the NILS-LSA, which were from the Japanese Wechsler Adult Intelligence Scale-Revised Short Forms (JWAIS-R-SF; Kobayashi et al., 1993). The revised WAIS (Wechsler, 1981) is a core component of many neuropsychological testing protocols. The four subtests used in the present study were as follows: Information, Similarities, Picture Completion and the Digit Symbol Substitution subtest.

1. Information: Participants were asked general knowledge questions about people, places and events (29 items, possible score range 0–29). This subtest measured the amount of factual knowledge.
2. Similarities: Participants were asked to state in what way two things are alike (14 items, possible score range 0–28). This subtest measured logical abstract reasoning.
3. Picture Completion: Participants were asked to spot the vital, missing element in a series of drawings (21 items, possible score range 0–21). This subtest is an expressive measure of knowledge, a nonverbal test of visual concentration, and it assesses long-term visual memory, which is an intellectual factor related to visualization ability, and the ability to differentiate essential from inessential details.
4. Digit Symbol Substitution: Participants were asked to write down the symbol that corresponded to a given number (as many as they could in 90 s, possible score range 0–93). This subtest measured processing speed, visual-motor coordination, visuospatial attention, executive cognitive function and working memory (Rosano et al., 2008).

Information and Similarities are verbal subtests, and load on the verbal-comprehension factor. Picture Completion and Digit Symbol Substitution are nonverbal tests. Picture Completion loads on the perceptual-organization factor. The Digit Symbol Substitution is a test of psychomotor speed and executive function (Wechsler, 1981).

In this study, trained examiners (clinical psychologists or psychology graduate students) administered the test to each participant on a one-to-one basis according to standard instructions. The details of the neuropsychological assessments in the NILS-LSA have been previously reported (Nishita et al., 2013).

### Audiometric Test and Other Measures

Air-conduction pure-tone thresholds at octave intervals from 0.5 kHz to 8 kHz were measured in a sound-proof booth by trained examiners using a standardized protocol and a diagnostic audiometer (AA-73A and AA-78; Rion, Tokyo, Japan). The pure-tone average threshold level of the better ear (PTABE) at frequencies of 0.5, 1, 2, and 4 kHz was used as an index of hearing status. The HI was defined as a PTABE >25 dB (which means bilateral hearing loss) according to WHO grades (WHO, 2012).

Participants filled out a series of questionnaires before the examination. Data of medical history of hypertension, diabetes, stroke, cardiac disease (1 = yes, 0 = no), current smoking status (1 = yes, 0 = no), marital status (1 = married, 0 = unmarried), occupation (1 = have an occupation, 0 = no occupation) and years of education (≤9, 10–12, 13–14, ≥15 years) were collected using self-report questionnaires. For the level of education, ≤9 refers to elementary school or junior high school, 10–12 refers to high school or junior high school under the former Japanese educational system, 13–14 refers to higher vocational school or junior college, and ≥15 years refers to college or graduate college.

### Statistical Analyses

Statistical analyses were conducted using the Statistical Analysis System (SAS) software version 9.3 (SAS Institute, Cary, NC, USA). Cross-sectional analyses were performed for the participants at baseline and longitudinally for the cumulative samples.

For univariate analysis, a *t*-test was used to assess differences of the continuous variables between the two groups, and comparisons of categorical variables were performed using the chi-square test. Unless otherwise noted, values are expressed as mean ± standard deviation (SD).

For analyses of the repeated measures of cognitive function, multivariable analyses of the cumulative data were conducted using a time-varying linear mixed-effects regression model with random intercepts (PROC MIXED procedure of SAS version 9.3), which is a generalized form of linear regression analysis that allows for repeated observations on each participant over time.

We estimated linear changes in the four neuropsychological subtests by HI status at baseline. The model used in the present analyses included fixed terms for the intercept (baseline performance for an individual with a value of zero on all predictors), HI (0 = no HI at baseline, 1 = HI at baseline), time (years elapsed since baseline), and a HI × time interaction term. As a combination of covariates, two models were analyzed. In Model 1, age (at baseline), sex, and education were adjusted. In Model 2, medical history of hypertension, diabetes, stroke, cardiac disease, current smoking status, marital status and occupation were used in addition to the covariates in Model 1. In order to control for practice effects of neuropsychological assessments, we added indicators of prior exposure to the tests. The procedure described by Alley et al. (2007) was used to account for the effects of repeated test exposure, by assigning the respondents 0 for baseline participation and 1 for each subsequent test administration.

Moreover, random effects of intercept (baseline performance) and slope (change over time) were calculated using an unstructured covariance matrix. The model-predicted 12-year change in cognitive abilities by baseline hearing status was estimated using the mean age (68.7 years) of the baseline samples taking into account potential confounders. The slopes were compared between the no-HI and HI groups.

## Results

The baseline demographics of study participants are shown in Table 1 by category of HI. According to the univariate analysis, significant differences between those with and without HI were observed in mean age, PTABE, proportion of male participants, educational level, comorbidity (i.e., diabetes and stroke) and the proportion of smokers. The baseline WAIS-R-SF profile by category of HI is presented in Table 2. The mean scores were significantly lower in those with HI than in those without HI, for all four cognitive subtests.

**Table 1:** **Baseline demographic profile by category of hearing impairment (HI)**.

	Total		No hearing impairment		Hearing impairment		*p*-value
	*N* = 1109		*N* = 698		*N* = 411		
Mean age, year	68.7 ± 5.5		67.2 ± 5.2		71.2 ± 5.0		<0.001
PTABE, dB	23.5 ± 12.6		15.7 ± 5.6		36.7 ± 10.0		<0.001
Sex, male [*n* (%)]	551	49.7	301	43.1	250	60.8	<0.001
Education [*n* (%)]
≤9 years	529	47.7	297	42.5	232	56.5	
10–12 years	400	36.1	275	39.4	125	30.4	<0.001
13–14 years	113	10.2	71	10.2	42	10.2
≥15 years	67	6.0	55	7.9	12	2.9	
Hypertension [*n* (%)]	414	37.3	259	37.1	155	37.7	0.840
Diabetes [*n* (%)]	128	11.5	64	9.2	64	15.6	0.001
Stroke [*n* (%)]	52	4.7	22	3.2	30	7.3	0.002
Cardiac disease [*n* (%)]	187	16.9	112	16.1	75	18.3	0.344
Smoker [*n* (%)]	198	17.9	104	14.9	94	22.9	0.001
Marital status, married [*n* (%)]	903	81.4	569	81.5	334	81.3	0.917
Occupation, have an occupation [*n* (%)]	338	30.5	225	32.2	113	27.5	0.098

*Abbreviations: PTABE, pure-tone average threshold level of the better ear at frequencies of 0.5, 1, 2, and 4 kHz; dB, decibel*.

**Table 2:** **Baseline profile of the Wechsler Adult Intelligence Scale-Revised Short Forms (WAIS-R-SF) subtests by category of hearing**.

	Maximum score	Total		No hearing impairment		Hearing impairment		*p*-value
		*N*		*N*		*N*	
Information	29	1108	12.7 ± 5.6	698	13.2 ± 5.6	410	11.8 ± 5.4	<0.001
Similarities	28	1107	10.9 ± 5.7	698	11.7 ± 5.7	409	9.5 ± 5.5	<0.001
Picture completion	21	1109	9.4 ± 3.7	698	9.8 ± 3.6	411	8.7 ± 3.8	<0.001
Digit symbol substitution	93	1106	40.9 ± 11.4	696	43.2 ± 11.3	410	37.2 ± 10.4	<0.001

The results of the multivariable analyses to assess the change in cognitive ability during the study period using a mixed-effects regression model are presented in Table 3. With respect to the main effect of HI on cognitive ability, there were significant associations of HI with scores in the four cognitive subtests. The significance was still observed even after adjusting for possible confounders in Model 2. In addition, time and age were significantly associated with scores in the four subtests when taking the potential confounders into account.

**Table 3:** **Relation of HI to change in the measure of cognitive ability during the study period according to a mixed-effects model**.

	Information				Similarities				Picture completion				Digit symbol substitution			
	Model 1^a^		Model 2^b^		Model 1^a^		Model 2^b^		Model 1^a^		Model 2^b^		Model 1^a^		Model 2^b^	
	*F*	*p* value	*F*	*p* value	*F*	*p* value	*F*	*p* value	*F*	*p* value	*F*	*p* value	*F*	*p* value	*F*	*p* value
Time	0.00	0.976	13.40	<0.001	1.81	0.179	4.31	0.038	99.97	<0.001	73.75	<0.001	165.78	<0.001	161.67	<0.001
Hearing impairment	8.67	0.003	6.49	0.011	12.12	0.001	9.63	0.002	8.30	0.004	6.96	0.008	6.99	0.008	4.70	0.030
Time × hearing impairment	8.09	0.005	10.60	0.001	2.03	0.155	2.28	0.131	1.66	0.198	1.51	0.219	10.26	0.001	11.29	0.001
Age	7.63	0.006	8.73	0.003	15.12	<0.001	16.41	<0.001	33.62	<0.001	37.45	<0.001	264.74	<0.001	255.45	<0.001

*Moderating variables. Model 1: Age at baseline, sex, and education. Model 2: Age at baseline, sex, education, medical history of hypertension, diabetes, stroke, and cardiac disease, and current smoking status, marital status, practice effects, and occupation*.

The HI × time (years) interaction was significant for the scores for Information (*p* = 0.005 in Model 1 and *p* = 0.001 in Model 2) and for Digit Symbol Substitution (*p* = 0.001 in Model 1 and *p* = 0.001 in Model 2), indicating that the decline over time differs between the group with HI and the group without HI.

**Figure 1:**
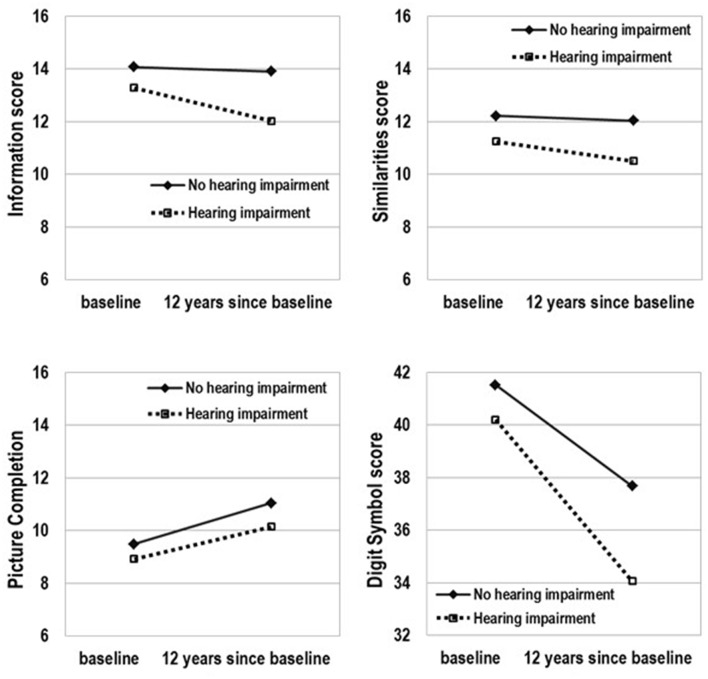
**Model-predicted 12-year change in cognitive ability by hearing status.** Adjusted for age, sex, education, medical history of hypertension, diabetes, stroke, and cardiac disease, and current smoking status, marital status, and occupation.

The model-predicted 12-year change in cognitive abilities by baseline hearing status that was estimated using the mean age (68.7 years) is presented in Figure 1 and Table 4. The slope was significant for all subtests in the HI group but it was not significant for Information and Similarities in the no-HI group. Furthermore, the significant difference in score decline between those with and without HI at baseline was observed for Information (*p* = 0.001) and Digit Symbol Substitution (*p* < 0.001). Over time, the scores of the HI group declined faster in the two subtests compared to those of the no-HI group. For Picture Completion, an improvement in the score was shown by both the HI and no-HI groups and there was no significant difference in slopes.

**Table 4:** **Model-predicted 12-year change in cognitive ability by hearing status**.

	Slope		Difference of slope	
		*p* value		*p* value
**Information**
Hearing impairment	−0.1049	<0.001		
			−0.0911	**0.001**
No hearing impairment	−0.0139	0.407
**Similarities**
Hearing impairment	−0.0610	0.030		
			−0.0460	0.131
No hearing impairment	−0.0150	0.420
**Picture completion**
Hearing impairment	0.1021	<0.001		
			−0.0271	0.219
No hearing impairment	0.1293	<0.001
**Digit symbol substitution**
Hearing impairment	−0.5120	<0.001		
			−0.1912	**<0.001**
No hearing impairment	−0.3209	<0.001

*Adjusted for age, sex, education, medical history of hypertension, diabetes, stroke, and cardiac disease, and current smoking status, marital status, practice effects, and occupation. Note: p-values in bold represent significance*.

## Discussion

In this population-based cohort study, elderly persons aged 60–79 years at baseline were followed for up to 13.3 years and were tested on their cognitive performance for a maximum of seven times. The change over time in cognitive performance differed significantly by hearing status at baseline, and the impact of HI on cognitive ability differed for the neuropsychological subtests.

As can be seen in Table 3, the presence of HI at baseline was associated with cognitive abilities independently from age, sex, educational level and other variables. Table 4 shows that the slope over time was significant for all subtests in the HI group after adjustment for potential confounders, which means that a 12-year change in this group was significant, regardless of the cognitive domain at least among those evaluated in the current analysis. In contrast, no significant decline was observed during the 12 years for Information and Similarities in the group without HI.

The time × HI interaction was statistically significant for the Information and Digit Symbol Substitution subtests, suggesting that there was a significant impact of HI on cognitive change over time. The model-predicted 12-year change indicated a significant difference in the declining slopes for the scores for Information and Digit Symbol Substitution between those with and without HI. The hearing-impaired elderly people showed a more rapid decline of the Information and Digit Symbol Substitution scores than their counterparts without HI. Some cognitive abilities appear more vulnerable to the aging effect than others, and vulnerability to the aging effect is likely to be modified by the presence of HI.

There have been many factor models that describe the structure of cognitive functioning. The CHC theory of cognitive abilities, which combines the Cattell-Horn Gf-Gc and Carroll three-stratum models, is one of the most widely accepted theories (Horn and Cattell, 1966; Carroll, 1993; Willis et al., 2011; Flanagan and Harrison, 2012; Schneider and Mcgrew, 2012).

“Fluid intelligence” represents the ability to use knowledge flexibly and adaptively and “crystallized intelligence” represents accumulated knowledge, which can be acquired throughout life via education and other life experiences (Craik and Bialystok, 2006). Aging does not negatively affect all cognitive abilities to the same degree. It is believed that fluid intelligence is vulnerable to the effects of aging whereas crystallized intelligence is robust (Kaufman and Horn, 1996), and that fluid intelligence declines from the mid-twenties onward whereas, crystallized intelligence rises until the age of 70 or so (Jones and Conrad, 1933; Welford, 1958).

The WAIS assesses a wide range of cognitive abilities and impairments. Factor analyses have documented four indices that define major cognitive domains: a verbal comprehension index, a perceptual organization index, a processing speed index and a working memory index (Manual WAIS-III WMS-III, 1997; Gläscher et al., 2009).

The Information and Similarities subtest scores relate to the verbal scales, which assess the amount of factual knowledge and logical abstract reasoning that are reflective of crystallized intelligence (Horn and Cattell, 1967; Cattell and Horn, 1978; Tulsky and Price, 2003; Kaufman and Lichtenberger, 2006). Studies addressing the association between WAIS scores and aging have demonstrated that the Information subtest has the topmost stability across the age ranges, and relatively high stability is observed in the Similarities subtest (Ryan et al., 2000; Hartshorne and Germine, 2015). In fact, the group without HI in the present study did not show the aging effect in the Information and Similarities subtests; however, this tolerance was lost in the group with HI.

Performance of the Digit Symbol Substitution subtest recruits different interrelated abilities. In order to perform well, several abilities (Yoran-Hegesh et al., 2009) are necessary such as perceptual speed (Stephens and Sreenivasan, 2002; Gilmore et al., 2004), motor speed, response selection, and shifting of attention as well as working memory (Kertzman et al., 2006). The Digit Symbol Substitution subtest has been defined as “one of the oldest and best established of all psychological tests” (Wechsler, 1958). The Digit Symbol Substitution scores in this study were extremely sensitive to the aging effect, consistent with the results of previous studies (Joy et al., 2004; Hartshorne and Germine, 2015). This sensitive effect was found in both groups with and without HI; however, the decline of scores over time was faster in the group with HI than in the group without HI. The vulnerability of the Digit Symbol Substitution performance to aging differs depending on the hearing status at baseline.

The cognitive system is multidimensional. As shown in Figure 1 and in the direction of the slope in Table 4, the scores of the Information, Similarities, and Digit Symbol Substitution subtests tended to decline over time, while there was a significant improvement in performance over time on the Picture Completion subtest. Since repeated administration of cognitive tests can improve performance, we controlled for practice effects of the neuropsychological tests. The practice effects may not be uniform across all cognitive domains.

The latest research focusing on the hearing-cognition relationship examines multiple cognitive domains using cross-sectional analyses. It is not currently known which aspects of cognition are closely related to hearing. In the UK Biobank, cognition is assessed using visually delivered measures of fluid intelligence, processing speed, executive function, number storage (Digit Span), and visuospatial working memory (Shape-pairs Matching). The broad cognitive categories tested in the UK Biobank have all been implicated in speech-in-noise hearing (Moore et al., 2014). In the Staying Keen in Later Life (SKILL) study in the USA, a battery of cognitive measures was used including the Mini-Mental State Examination (MMSE), speed of processing (i.e., Digit Symbol Substitution and Copy, Trail Making Test Part A, Letter and Pattern Comparison and Useful Field of View), executive function (i.e., Trail Making Test Part B, Stroop Color-Word Interference Task), and memory (i.e., Wechsler Memory Scale-III Digit and Spatial Span, Hopkins Verbal Learning Test). The three-frequency PTABE accounted for a significant amount of variance in the MMSE and all cognitive outcomes except for in the Trail Making Test Part A (Harrison Bush et al., 2015). In the Busselton Healthy Ageing Study of Australia, the Cognitive Drug Research computerized assessment system was used and cognitive scores were derived by factor analysis: namely, for quality of episodic secondary memory, quality of working memory, speed of memory, continuity of attention and power of attention. In their results, hearing loss according to pure-tone averages was not a predictor of cognitive performance in any domain (Bucks et al., 2016).

Although there is a gradual increase in the number of cross-sectional analyses, to the best of our knowledge, the present study is the only one that has longitudinally analyzed the association between multiple cognitive domains and hearing in elderly people.

Several theories of the possible mechanisms of the hearing-cognition relationship have been proposed as follows: (1) the sensory deprivation hypothesis, which argues that perceptual decline causes more permanent cognitive decline; (2) the information degradation hypothesis; (3) the cognitive-load-on-perception hypothesis, which suggests that impaired encoding by the cochlea may require extra cognitive processing effort, limiting the effort available for encoding the content of speech into memory (this increasing cognitive load due to HI has been termed *effortful listening*; Deal et al. (2015)); and (4) the common-cause hypothesis, in which a third factor causes decline in both hearing and cognition (Committee on Hearing, 1988; Harrison Bush et al., 2015; Wayne and Johnsrude, 2015).

We previously reported the association of education level with the degree of cognitive change during a follow-up of approximately 10 years in the NILS-LSA. Contrary to expectations, individuals with higher levels of education experienced a rather greater decline in the Digit Symbol Substitution subtest scores than those with less education. On the other hand, in the present analysis, individuals with HI demonstrated a greater decline in the Digit Symbol Substitution subtest scores over time than those without HI. It is hoped that intervening in HI will prevent the effect on cognitive decline. Some research studies have demonstrated that hearing-aid use attenuates such cognitive decline (Amieva et al., 2015). On the other hand, according to Lodeiro-Fernández et al. (2015) reduced hearing level partially explains verbal comprehension performance only in the group with less cognitive impairment, that means the normal/predementia group. When cognitive impairment increases (moderate/moderately severe dementia group), the importance of HI on language function decreases. They implied that the correction of HI by the use of hearing aids would not significantly ameliorate the cognitive language dysfunction observed in the dementia group. Further investigation is needed, and we remain ready to assess an intervention in research.

In conclusion, we investigated whether baseline hearing status was associated with the degree of cognitive change assessed with four neuropsychological subtests during a 12-year follow-up of an elderly Japanese population. The rate of change over time in cognitive performance differed significantly depending on the presence or absence of HI at baseline. The scores of the HI group declined faster in the Information and Digit Symbol Substitution subtests compared to those in the no-HI group. Moreover, in the individuals without HI at baseline, no significant decline was observed during the 12 years for the Information and Similarities subtests. In contrast, the slope for the HI group was significant for all subtests. The current results imply that the presence of HI could be a potential modifier of cognitive decline and that intervening in HI raises hope for the preventive effect on cognitive decline for some aspects of cognitive ability.

## Author Contributions

YU: drafted the manuscript, and took responsibility for the accuracy of the data analysis. YU, YN, CT, SS, RO, FA and HS: contributed to the collection of data and statistical analyses. HS and FA: contributed to the study concept and design of NILS-LSA. HU and TN: supervised the data collection. YU, SS, TN and HS: obtained funding. All the authors contributed to the interpretation of the data and approved the final version of the manuscript before submission and approved the final version to be published.

## Sponsor’S Role

The funding sources had no role in the study design or conduct of the study; in the collection, management, analysis, or interpretation of the data; in the writing of the manuscript; or the decision to submit.

## Funding

This study was supported in part by the Japan Society for the Promotion of Science KAKENHI (Grant Nos. 26502016 and 16H03264), the Research Grant for Longevity Sciences (Grant Nos. 20shi-2 and 21A-17), and by the Ministry of Health, Labour and Welfare of Japan (Grant No. 25-2).

## Conflict of Interest Statement

The authors declare that the research was conducted in the absence of any commercial or financial relationships that could be construed as a potential conflict of interest. The reviewer KRB and handling Editor declared their shared affiliation, and the handling Editor states that the process nevertheless met the standards of a fair and objective review.
